# Personality and Performance Amongst Athletes: A Systematic Review

**DOI:** 10.7759/cureus.96149

**Published:** 2025-11-05

**Authors:** Auston Hsieh, Mahalaxmi Some, Pradeep Vanguri

**Affiliations:** 1 Department of Medicine, Halmos College of Arts and Sciences, Nova Southeastern University, Fort Lauderdale, USA; 2 Department of Health and Human Performance, Dr. Kiran C. Patel College of Osteopathic Medicine, Nova Southeastern University, Fort Lauderdale, USA

**Keywords:** athletic performance, big five personality test, dark triad, perfectionism, personality

## Abstract

Athletic performance is shaped by both physical and environmental factors, yet an athlete’s personality can also play a significant role. The Big Five personality test contains common traits used to characterize the human personality, and it evaluates openness, conscientiousness, agreeableness, extraversion, and neuroticism. The Big Five traits influence how athletes think, behave, and perform, while the Dark Triad traits and perfectionism may also impact performance. The Dark Triad consists of narcissism, Machiavellianism, and psychopathy, three common negative personality traits. Perfectionism consists of adaptive and maladaptive perfectionism. This meta-analysis examines the relationship between personality and athletic performance by conducting a literature search on PubMed. The search terms “personality” and “athletic performance” yielded 144 studies. Studies not within the last 10 years were excluded, and studies were filtered to only include primary articles and correct study design. As a result, 22 studies were included in the meta-analysis. Conscientiousness and extraversion predicted higher performance, openness and agreeableness had mixed impacts, while neuroticism was linked to poorer outcomes. Dark Triad traits, specifically narcissism, were shown to enhance performance in competitive settings, while Machiavellianism had mixed impacts, and psychopathy led to worse performance. Perfectionism revealed a dual effect depending on the type. The impacts of personality traits on performance in athletes are not inherently positive or negative but instead depend on many factors, including the type of sport, competition level, and more. Future research should address these limitations to better encompass the confounding variables.

## Introduction and background

Athletic performance is influenced not only by a wide variety of physical and environmental factors but also by psychological factors, such as personality, that play a critical role in shaping an athlete’s performance [1]. An athlete’s personality can affect how they train, interact with teammates, respond to setbacks, and become a leader on and off the field. In many cases, personality traits help distinguish elite athletes from amateur athletes [2]. 

One of the most influential frameworks to assess personality is the Big Five personality test, which emerged in the mid-20^th^ century from decades of factor-analytic research aimed at capturing the fundamental dimensions of human personality. The model organizes personality into five traits: openness (creativity and adaptability), conscientiousness (discipline and reliability), agreeableness (cooperativeness and empathy), extraversion (sociability and assertiveness), and neuroticism (emotional instability and proneness to negative affect) [3]. The Big Five’s enduring popularity in the field of psychology stems from its cross-cultural validity and empirical evidence to explain a wide range of human behaviors, making it known as a “gold standard” for personality assessment.

Beyond the Big Five, there are other analysis tests that capture the darker and more complex aspects of personality. The Dark Triad, a framework introduced in the early 2000s, examines narcissism (arrogance and sense of entitlement), Machiavellianism (manipulation and deceit), and psychopathy (impulsivity and lack of empathy)-all traits that are known to be socially maladaptive yet may sometimes benefit competitive performance [4]. For instance, narcissism may fuel confidence under pressure, while Machiavellian characteristics could manifest into strategic thinking in high-pressure situations. Another relevant construct to be included is perfectionism, which exists in both an adaptive and maladaptive form. Adaptive perfectionism is known to drive discipline and high standards, while maladaptive perfectionism can lead to burnout and anxiety [5].

The existing literature on the psychological aspect of performance remains fragmented. Many studies examine specific groups, such as amateur or collegiate athletes, while others focus exclusively on either the Big Five or non-Big Five traits. As a result, this leads to a lack of integrated understanding of how personality contributes to performance across different sports, competitive levels, and team environments. By identifying the traits that consistently support or hinder athletic performance, the key players themselves will be able to implement targeted mental performance strategies and develop a better training environment. 

The purpose of this study is to conduct a meta-analysis of peer-reviewed research published over the last decade to examine the relationship between personality traits and athletic performance. This study aims to bridge the gap between fragmented findings by evaluating both traditional and emerging personality constructs across a variety of different sports and athlete levels. Such insights will be able to provide a clearer analysis of which specific personality traits enhance performance, worsen it, or have conditional effects, and have the opportunity to refine training environments and develop targeted mental performance strategies for athletes. Understanding the personality traits’ influence on specific sports and competition levels can help coaches, trainers, and psychologists maximize athletes’ performance and well-being.

## Review

Methods

**Figure 1:**
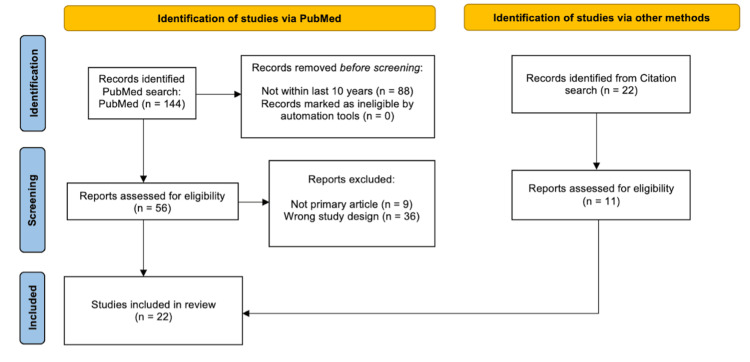
A flow diagram of studies identified via PubMed and other methods

A systematic evaluation of peer-reviewed published articles within the last decade was conducted to determine if there are certain personality aspects linked to performance. Peer-reviewed published articles from the PubMed database were analyzed using the search terms “personality” and “athletic performance” with the Boolean operator "AND", which yielded 144 studies. Studies were excluded if they were not published within the past 10 years, reducing the total number of usable studies to 56. Studies were filtered to only include primary articles and correct study design, which investigate the impact of personality traits on the performance of athletes. This search concluded with a total of 11 studies. A citation search was conducted in filtered studies to identify more eligible studies. The citation search added 11 studies for a total of 22 studies. The screening process to determine the eligibility of the studies for this meta-analysis was followed by utilizing the flow diagram shown in Figure 1. 

The Big Five traits

The Big Five personality traits have been variously explored to see how the particular traits influence athletic performance across different sports and competition levels. The table below summarizes the key points found in articles that focused on the Big Five personality traits in athletes’ performance. Across multiple studies, conscientiousness consistently emerged as a strong positive predictor of athletic success, mostly in team sports [6,7], while extraversion and openness to experience are also frequently associated with improved performance, particularly in elite athletes [8,9]. While agreeableness also showed some positive association with performance, the role of this particular trait appears to be more context-dependent [10,11]. Table 1 addresses the key points relating to the Big Five traits that will be discussed throughout this review. 

**Table 1:** Summary points of articles on the Big Five traits

Author(s), Year	Title	Summary Points
Conway, 2016 [6]	Investigating the relationship between personality traits and athletic performance among elite hockey players	Conscientiousness and agreeableness led to better hockey performance. While neuroticism led to worse performance. Extraversion significantly predicted games played.
Mckenzie, 2021 [7]	Functional Effects of Personality on Individual and Team Sport Success	In collegiate team sports, conscientiousness and openness are the two prominent personality traits leading to athletic success.
Vaughan & Madigan, 2020 [8]	Decoding the elite soccer player's psychological profile	Elite soccer players have elevated levels of conscientiousness, extraversion, and openness to experience. They had reduced levels of neuroticism and agreeableness.
Li et al., 2024 [9]	Exploring performance of athletic individuals: Tying athletic behaviors and big-five personality traits with sports performance	Big Five personality traits are significant predictors of athletic behaviors; neuroticism is found to be insignificant. The role of athletic behavior in the nexus between personality traits and sports performance is also significant.
Habib et. al., 2019 [10]	Personality Traits Predict in Sports Performance among University Athletes	Extraversion, agreeableness, conscientiousness, and openness were positively correlated with team sports performance. While neuroticism was negatively correlated.
Chen et al., 2021 [11]	Personality Traits, Loneliness, and Affect Among Boxers.	Neuroticism had a negative effect, while conscientiousness, extraversion, and agreeableness had a positive effect on boxers. Openness had no effect on boxers.
Bonetti et al., 2025 [12]	Effects of Feedback Type and Personality on 2,000-m Ergometer Performance in Female Varsity Collegiate Rowers	Neuroticism has a negative impact on 2,000-ergometer performance, while conscientiousness and agreeableness have a positive impact.
Piepiora, 2021 [13]	Assessment of Personality Traits Influencing the Performance of Men in Team Sports in Terms of the Big Five	Champions of elite team sports were characterized by a lower level of neuroticism, a higher level of extraversion, and openness to experiences in relation to other sportsmen. It was also confirmed that the personality traits distribution levels depend on the sports discipline.

The Dark Triad traits

The Dark Triad includes personality traits such as Machiavellianism, narcissism, and psychopathy, and it presents an overall nuanced view in relation to athletic performance. Several studies suggest that these traits may possibly enhance specific aspects of performance, particularly when present in competitive settings. For instance, narcissism is positively correlated to mental toughness and achievement-oriented values [14,15], while all three traits have been linked to improved task performance, such as basketball free-throw accuracy [16] and overall competitiveness [17]. Some results even support a positive effect on general sports performance, especially when an athlete’s focus is aligned with achievement [18]. However, other studies revealed limited or no direct performance benefit. [19] found no correlation between the Dark Triad and winning percentage in professional fighters, and [5] observed no elevation in anxiety, anger, or depression among mixed martial artists compared to non-fighters. The variety in the findings of the studies suggests that while the Dark Triad traits are present in athletes, especially professionals, they are more likely to enhance competitive drive and mental resilience than directly predict performance outcomes, and their influence on athletic performance may vary across sports and metrics. Table 2 addresses the key points relating to the Dark Triad traits that will be discussed throughout this review. 

**Table 2:** Summary points of articles on the Dark Triad traits

Author(s), Year	Title	Summary Points
Pino et al., 2022 [5]	Trait Anxiety in Mixed Martial Arts	The mixed martial artists did not have higher anxiety, anger, or depression compared to non-fighters.
Vaughan et al., 2018 [14]	Harder, better, faster, stronger? Mental toughness, the dark triad and physical activity	Professional athletes had higher levels of the Dark Triad traits than amateur athletes and non-athletes. There is a positive relationship between narcissism and mental toughness.
Caliskan & Özer, 2019 [15]	Relationship between dark triad personality traits and work values among athletes	In athletes, narcissism is correlated with prestige and achievement, while Machiavellianism and psychopathy don’t lead to creativity and achievement.
Wu et al., 2021 [16]	The winner takes it all: The mediating role of competitive orientations in the Dark Triad and sport task performance relationship	The Dark Triad traits, which are Machiavellianism, narcissism, and psychopath, lead to better basketball free-throw performance.
González-Hernández et al., 2020 [17]	Why Negative or Positive, If It makes Me Win? Dark Personality in Spanish Competitive Athletes	Athletes’ competitiveness is strongly related to Machiavellianism, narcissism, and psychopathy.
Adavize et al., 2024 [18]	Influence of dark triad on sports performance of athletes in Prince Abubakar Audu University Anyigba Kogi State	Dark Triad traits were seen in athletes, and they had a positive impact on sports performance. The traits must be focused on achievement in sports.
Lundkvist et al., 2021 [19]	The Dark Triad and Professional Fighters: Destigmatizing Male Combat Athletes	There is no significant correlation between winning percentage for professional fighters and Machiavellianism, psychopathy, or narcissism.

Perfectionism

The literature presented on perfectionism in athletes highlights a complex duality between its adaptive and maladaptive forms. Perfectionistic strivings, or the desire to excel and meet high standards, have been consistently correlated with enhanced athletic performance, especially when coupled with effective coping strategies [20,21,22]. Athletes who view perfectionism as a challenge rather than a threat tend to perform better, even under adverse conditions such as poor sleep [21,22]. On the other hand, perfectionistic concerns, the fear of failure, or excessive worry over mistakes have shown no consistent benefit to performance and are often associated with negative psychological and physical outcomes. This includes burnout in junior athletes [23], increased vulnerability to the Female Athlete Triad [24], and greater sensitivity to parental pressure [23]. However, in some cases, maladaptive perfectionism even coexisted with reduced injury time [25], though these findings require cautious interpretation. Overall, the literature suggests that adaptive perfectionism can serve as a performance enhancer, while maladaptive perfectionism poses mental and physical health risks and essentially underscores the importance of psychological support and goal reframing in competitive sport settings. Table 3 addresses the key points relating to perfectionism that will be discussed throughout this review. 

**Table 3:** Summary points of articles on perfectionism

Author(s), Year	Title	Summary Points
Mallinson-Howard et al., 2020 [20]	A three-sample study of perfectionism and field test performance in athletes	Striving for perfection led to better performance. Negative reactions to imperfection were unrelated to performance.
Meng et al., 2024 [21]	How the perfectionistic climate of a sports team predicts the athletic performance of elite athletes: a case study of the CUBAL women’s basketball team	Perfectionism boosts performance when it is seen as a challenge with good coping strategies. However, it harms performance if it is seen as a threat with bad coping strategies.
Roy et al., 2023 [22]	The relationship between sleep, perfectionistic strivings, perfectionistic concerns, and academic and sports performance in young athletes	Athletes with high levels of perfectionist strivings still performed well despite poor sleep habits. Perfectionistic concerns had no effect on athlete performance.
Gustafsson et al., 2015 [23]	Profiles of perfectionism, parental climate, and burnout among competitive junior athletes	Junior athletes high in perfectionism may be at greater risk for burnout, especially the case in perceiving their parents' concerns about failure and winning
Brooks et al., 2024 [24]	Impact of Perfectionism on the Risk of the Female Athlete Triad in Collegiate Athletes	Maladaptive perfectionism was linked to increased risk of the female athlete triad. While adaptive perfectionism was not related to triad risk.
Gil-Caselles et al., 2025 [25]	A perfectionism, mental health and vulnerability to injury in triathletes	In amateur athletes, having high maladaptive perfectionism was linked to decreased time that athletes were out due to injury.
Nam & Han, 2020 [26]	The Comparison of Perfectionism and Commitment between Professional and Amateur Golfers and the Association between Perfectionism and Commitment in the Two Groups	Golfers seeking to attain high levels of performance must consider the mental aspect of golfing and find ways to maximize commitment levels while minimizing perfectionist traits.

Traits that improve performance

Conscientiousness, defined as being diligent and responsible, and extraversion, characterized by outgoing and energetic behavior, are both significant predictors of athletic performance [3,9]. Studies have shown that conscientiousness and extraversion in university athletes in team sports led to athletic success and enhanced sports performance [7,10]. In boxers, conscientiousness and extraversion have a positive effect [11]. However, in elite hockey players and collegiate rowers, only conscientiousness consistently predicted stronger outcomes [6,12]. This pattern suggests that while being outgoing may lead to success in early or less structured training environments, long-term or elite-level performance relies more heavily on internal drive and goal-oriented drive. In other words, enthusiasm and sociability may provide an initial advantage, but the sustained edge belongs to athletes who are methodical and resilient. 

The role of extraversion appears to shift depending on the sport type and competitive level. Athletes who are also talkative and assertive may perform well in individual sports, while their impact on performance in collegiate team sports ranges from beneficial to negligible. In team sports, elite soccer players had elevated conscientiousness and extraversion [8]. However, champions of elite team sports only had elevated levels of extraversion [13]. In the higher level of sports, including collegiate and elite, the positive effects of extraversion seem to diminish while conscientiousness remains significant. As athletes advance, the need for discipline becomes more dominant, but at the very highest levels of extraversion, the ability to inspire and energize others regains importance. 

Similarly, narcissism, one of the Dark Triad traits, also shows a surprising connection to improved performance, being displayed through mental toughness and resilience. Multiple studies report that narcissism is correlated with mental toughness, prestige-oriented values, and task performance, particularly in individual-focused sports [14,15,16]. This trait fosters resilience under pressure and a strong sense of self-belief, which are both critical in high-stakes environments in athletics. For instance, narcissism has been linked to improved free-throw shooting in basketball players and greater achievement orientation among athletes in general [15,16]. Furthermore, professional athletes were found to exhibit significantly higher levels of narcissism in comparison to amateur and non-athletes, which in turn supports the idea that this trait may give competitors a psychological “edge” at the elite level [14]. Despite these potential issues, narcissism’s link to individual drive and competitive success reinforces its utility in improving and enhancing athletic performance. 

Finally, the pattern of goal-oriented resilience and internal drive is also what maintains adaptive perfectionism, or perfectionistic strivings, as a powerful enhancer of athletic performance. It is described as being the most positively supported aspect of perfectionism in athletic performance. Athletes who are able to channel their perfectionistic tendencies through goal setting and self-discipline have a desire for excellence and tend to perform better, especially when combined with effective coping strategies and a positive perception of challenge. Multiple studies have shown that striving for perfection leads to enhanced field test performance, sustained athletic achievement, and even maintained success under suboptimal conditions, such as poor sleep [20,21,22]. These findings suggest that when perfectionism is driven by internal motivation rather than fear, it is a very effective performance enhancer, much like the self-discipline of conscientiousness and the confidence of narcissism. Athletes who self-reflect and embrace challenges transform perfectionism into a tool for resilience, whereas those without this balance risk burnout. 

Traits with mixed or conditional impacts

While some personality traits generally enhance performance, others, such as openness to experience and agreeableness, show mixed or context-dependent effects. Openness to experience, marked by curiosity and creativity, and agreeableness, defined by cooperativeness and empathy, are both significant predictors of athletic performance [3,9]. Studies have shown that these traits in university athletes in team sports led to athletic success and enhanced sports performance [7,10], and agreeableness has been linked to stronger performance in elite hockey players, boxers, and rowers [6,11,12]. These findings suggest that while openness supports adaptability to strategy in many sports, its benefits do not always translate to highly individual closed-skill sports. In team sports, elite soccer players and champions of elite team sports had elevated levels of openness [8,13]. However, elite soccer players had reduced levels of agreeableness [8]. At the highest level of sports, elite athletes tend to care less about pleasing teammates and instead are more assertive. They are open to new tactics and strategies with the main goal of competing to win. 

Machiavellianism describes manipulation, strategic thinking, and a self-interested focus. In the context of athletics, the studies described in this meta-analysis have shown mixed outcomes regarding their influence on performance. Machiavellianism has been positively correlated with competitiveness and a drive to win, especially in highly competitive sports settings like basketball and elite competitions [16,17]. However, Lundkvist et al. [19] found no significant correlation between Machiavellianism and actual winning percentages among professional fighters. This suggests that while Machiavellian athletes may excel in strategic thinking, the translation of this trait into performance appears to be extremely conditional, varying by sport and whether interpersonal manipulation can be effectively leveraged or, in the end, becomes detrimental to a team dynamic.

Traits that detract from performance

Neuroticism, defined by tendencies toward anxiety, anger, and negative emotions, is one of the traits strongly associated with worse outcomes. While some models suggest it is an insignificant predictor of athletic performance [3,9], several studies in the meta-analysis have shown that neuroticism in university athletes in team sports did not lead to athletic success, instead leading to worse sports performance [7,10]. Specifically, neuroticism in elite hockey players, boxers, and collegiate rowers was associated with decreased athletic success [6,11,12]. Athletes who struggle to regulate their emotions often experience mental distractions, lowered concentration, and poor stress management during competition. These negative emotional states can lead to performance breakdowns at crucial moments. In contrast, elite soccer players exhibited reduced levels of neuroticism [8], underscoring how emotional stability supports resilience at the top level. The ability to remain optimistic and composed, even in high-pressure situations, appears to be a defining trait among top-performing athletes. 

Some studies have shown that psychopathy is related to increased competitiveness and task performance, similar to that of Machiavellianism and narcissism [16,17]. Exhibiting psychopathy as an athlete describes a fearless, risk-taking style that can benefit performance in high-contact or combat sports, but other bodies of literature indicate no significant correlation between psychopathy and winning outcomes, as seen in professional fighters [19]. Furthermore, psychopathy was not linked to creativity or achievement-oriented values in athletic work settings [15]. Thus, psychopathy may offer short-term benefits in niche sports; its overall impact on performance is neutral at best and detrimental in most athletic environments. 

Unlike adaptive perfectionism, which is internally driven and associated with resilience, maladaptive perfectionism stems from a fear of failure, where an athlete is overly self-critical and internalizes external pressures, usually from parents or coaches. Gustafsson et al. [23] found that junior athletes high in perfectionistic concerns were at greater risk for burnout, specifically when they perceived a parental climate focused on winning and “avoiding failure at all costs”. Similarly, Brooks et al. [24] linked maladaptive perfectionism to an increased risk of the Female Athlete Triad, which is a serious health condition caused by disordered eating, menstrual dysfunction, and bone density loss in female athletes. These findings highlighted both the psychological and physiological toll that perfectionistic concerns can take when not managed. While perfectionism in its adaptive form has the ability to build resilience and foster success, its maladaptive counterpart creates a fragile and high-pressure mindset that ultimately undermines both performance and health.

Limitations and future directions

This meta-analysis included athletes across a wide range of competitive levels, from amateurs to elite athletes. While this scope provides a broad overview, it also makes it difficult to determine whether certain traits operate differently depending on the level of competition. For example, traits like extraversion may be more useful for team cohesion among amateurs but less relevant at elite levels, where discipline and skill precede other factors. Second, performance in individual sports often depends more on traits such as resilience and self-confidence, whereas team sports place greater emphasis on cooperation and communication. Finally, methodological variation across the studies used in this analysis presents a challenge. Different personality tests were used to measure the same constructs, each with slightly different definitions and scales. Future research should move toward greater standardization by relying on validated, widely used instruments, such as the Big Five or Dark Triad scales, improving comparability and allowing for stronger meta-analytic conclusions.

## Conclusions

The impacts of personality traits on performance in athletes are not inherently positive or negative but instead depend on many factors, including the type of sport, competition level, and more. Although sports can be characterized as solo or team sports, some personality traits were beneficial or detrimental depending on the sport. With respect to competition level, some traits were only advantageous at the lower levels but diminished in impact at the higher levels, while others demonstrated the opposite pattern. Understanding the link between personality traits and the influence they have in specific sports and competition levels is beneficial for coaches to tailor training plans and for sports psychologists to implement interventions to maximize their performance and well-being. Further research is crucial to better understand the personality's influence on specific sports and competition levels.

## References

[1] Calleja-González J, Mallo J, Cos F (2022). A commentary of factors related to player availability and its influence on performance in elite team sports. Front Sports Act Living.

[2] Shuai Y, Wang S, Liu X, Kueh YC, Kuan G (2023). The influence of the five-factor model of personality on performance in competitive sports: a review. Front Psychol.

[3] Piepiora PA, Čaplová P, Zimoń P, Gumienna R (2024). On research into the relationship between personality traits and the sporting level of competitive, professional and elite athletes. Front Psychol.

[4] Yang JH, Yang HJ, Choi C, Bum CH (2024). Relationship between athletes' Big Five Model of personality and athletic performance: meta-analysis. Behav Sci (Basel).

[5] Pino J, Hauwermeiren OV, Kwamanakweenda J, Peacock C, Tartar J (2022). Trait anxiety in mixed martial artists. Neuro Sports.

[6] Conway BH (2016). Investigating the relationship between personality traits and athletic performance
among elite hockey players. EWU Masters Thesis Collection.

[7] Mckenzie AI (2021). Functional effects of personality on individual and team sport success. University of Windsor.

[8] Vaughan RS, Madigan DJ (2021). The winner takes it all: the mediating role of competitive orientations in the Dark Triad and sport task performance relationship. Eur J Sport Sci.

[9] Li Q, Xiao D, Zeng Q (2024). Exploring performance of athletic individuals: tying athletic behaviors and big-five personality traits with sports performance. PLoS One.

[10] Habib MB, Waris S, Afzal S (2019). Personality traits predict in sports performance among university athletes. Spark.

[11] Chen X, Qiu N, Chen C, Zhai L (2021). Personality traits, loneliness, and affect among boxers. Front Psychol.

[12] Bonetti L, Vestberg T, Jafari R (2025). Decoding the elite soccer player's psychological profile. Proc Natl Acad Sci U S A.

[13] Piepiora P (2021). Assessment of personality traits influencing the performance of men in team sports in terms of the Big Five. Front Psychol.

[14] Vaughan R, Carter GL, Cockroft D, Maggiorini L (2018). Harder, better, faster, stronger? Mental toughness, the dark triad and physical activity. Pers Individ Differ.

[15] Caliskan G, Özer A (2019). Relationship between dark triad personality traits and work values among athletes. Curr Psychol.

[16] Wu CH, Nien JT, Lin CY (2021). Relationship between mindfulness, psychological skills, and mental toughness in college athletes. Int J Environ Res Public Health.

[17] González-Hernández J, Cuevas-Campos R, Tovar-Gálvez MI, Melguizo-Rodríguez L (2020). Why negative or positive, if it makes me win? Dark personality in Spanish competitive athletes. Int J Environ Res Public Health.

[18] Sunday FA, Olukogbe OO, Umar IF (2024). Influence of dark triad on sports performance of athletes in Prince Abubakar Audu University Anyigba Kogi State. J Health Hum Mov Stud.

[19] Lundkvist E, Gustafsson H, Björklund G, Davis P, Ivarsson A (2021). Relating competitive golfers’ perceived emotions and performance. Percept Mot Skills.

[20] Mallinson-Howard SH, Madigan DJ, Jowett GE (2021). A three-sample study of perfectionism and field test performance in athletes. Eur J Sport Sci.

[21] Meng M, Su RH, Kogiso K (2024). How the perfectionistic climate of a sports team predicts the athletic performance of elite athletes: a case study of the CUBAL women's basketball team. Front Psychol.

[22] Roy J, Godin R, Gaudreault P, Forest G (2023). The relationship between sleep, perfectionistic strivings, perfectionistic concerns, and academic and sports performance in young athletes. Chronobiol Int.

[23] Gustafsson H, Hill AP, Stenling A, Wagnsson S (2016). Profiles of perfectionism, parental climate, and burnout among competitive junior athletes. Scand J Med Sci Sports.

[24] Brooks SG, Peach HD, Howden R, Lowrie J, Marino JS (2025). Impact of perfectionism on the risk of the female athlete triad in collegiate athletes. J Strength Cond Res.

[25] Gil-Caselles L, Barquín RR, Gimenez-Egido JM, García-Naveira A, Olmedilla A (2025). A perfectionism, mental health and vulnerability to injury in triathletes. Front Psychol.

[26] Nam JJ, Han DH (2020). The comparison of perfectionism and commitment between professional and amateur golfers and the association between perfectionism and commitment in the two groups. Int J Environ Res Public Health.

